# Isolation and characterization of an ammonium-oxidizing iron reducer: *Acidimicrobiaceae* sp. A6

**DOI:** 10.1371/journal.pone.0194007

**Published:** 2018-04-11

**Authors:** Shan Huang, Peter R. Jaffé

**Affiliations:** Department of Civil and Environmental Engineering, Princeton University, Princeton, New Jersey, United States of America; The University of Akron, UNITED STATES

## Abstract

*Acidimicrobiaceae* sp. A6 (ATCC, PTA-122488), a strain that has been previously reported to play a key role in the oxidation of ammonium (NH_4_^+^) under iron reducing conditions, has now been isolated from riparian wetland soils in New Jersey, USA. Incubations of this strain in a medium containing ferrihydrite as the ferric iron [Fe(III)] source, CO_2_ as the carbon source, under room temperature, and a pH of 4.5, resulted in 52% of NH_4_^+^ removal over a 20-day incubation period, while reducing Fe(III) in the expected stoichiometric ratio when NH_4_^+^ was oxidized to nitrite with Fe(III) as the electron acceptor. This study demonstrates that this new isolated strain is capable of oxidizing NH_4_^+^ while reducing iron under anaerobic conditions.

## Introduction

Ammonium (NH_4_^+^) can be oxidized in the presence of dissolved oxygen (DO) to nitrite (NO_2_^-^) by either ammonia oxidizing bacteria (AOB) or ammonia oxidizing archaea (AOA) [1, 2]. While AOB are likely to oxidize the bulk of NH_4_^+^ in aerobic environments, AOA can oxidize NH_4_^+^ in the presence of much lower DO levels [2, 3]. NH_4_^+^ can also be oxidized by anammox bacteria in the absence of oxygen, with NO_2_^-^ as the electron acceptor [2]. In addition to these well-established pathways, a recently discovered process of anaerobic NH_4_^+^ oxidation coupled to iron reduction in the absence of either oxygen, nitrate (NO_3_^-^), or NO_2_^-^ has received significant interest. The process was first noted in a forested riparian wetland in New Jersey [4–6], and was also observed in a biological reactor and coined Feammox [7]. The Feammox process has been reported so far in riparian wetland soils in New Jersey [4–6, 8], in tropical rainforest soils in Puerto Rico [9], in wetland soils in South Carolina [8], and at various forested and wetland locations in Southern China, including paddy soils [8].

Recent studies indicate that the Feammox reaction appears to be more common in acidic, iron rich, wetland environments [4, 8–10]. In our previous study, conducted with soil samples obtained from a forest riparian wetland (Assunpink Wildlife Management Area, New Jersey), simultaneous anaerobic NH_4_^+^ oxidation and iron reduction reactions was always accompanied by the increase in abundance of a group of *Actinobacteria*. Among them, an *Acidimicrobiaceae*-like bacterium, which was first found via PCR-DGGE analyses, allowing for its 16S rRNA gene sequencing, was labeled uncultured *Acidimicrobiaceae* bacterium A6 [4]. This *Acidimicrobiaceae* bacterium, a previously unreported one in the *Acidimicrobiaceae* family has been enriched in batch clutures and be identified as playing a key role in the Feammox process. The stoichiometry and change in free energy for the Feammox reaction when the ferric iron (Fe(III)) source is ferrihydrite was given by Huang and Jaffé [6, 8] for their experimental conditions (pH = 4, NO_2_^-^ = 0.5 mmol/l, Fe(II) = 1×10^−6^ mmol/l) as:

$$3\;Fe_{2}O_{3}\cdot 0.5H_{2}O+10H^{+}+NH_{4}^{+}\to \;6Fe^{2+}+8.5\;H_{2}O+NO_{2}^{-}$$ (1)

Where Δ*G*_*r*_ ≤—145.08 *kJ mol*^*-1*^. This equation indicates that soils with bioavailable Fe(III) phases, low pH, and conditions that prevent the buildup of dissolved Fe(II) and NO_2_^-^ should be favorable for the Feammox process, and that this reaction could possibly be an autotrophic iron reduction process [4]. Incubations with *Acidimicrobiaceae* enrichment cultures from the Assunpink site have shown that acetylene did block the reduction of nitrous oxide to N_2_ but did not block the Feammox reaction. These incubations also showed that the amount (in moles) of NH_4_^+^ removed was equal to that of NO_2_^-^ + nitrous oxide (N_2_O) accumulated [4].

Demonstrating unequivocally that this *Acidimicrobiaceae*-like bacterium A6 is capable of oxidizing NH_4_^+^ while reducing iron requires its isolation. Hence, the objective of this research was to isolate this *Acidimicrobiaceae* bacterium from the Feammox enrichment culture and verify that the pure strain is capable of oxidizing NH_4_^+^ while reducing iron under various environmental conditions.

## Methods and materials

### Enrichment feammox culture in batch experiments

Soil samples from the forested riparian wetland in New Jersey (Assunpink Wildlife Management Area) mentioned above, where we have previously observed NH_4_^+^ oxidation under iron reducing conditions [4, 5, 6, 8], were collected during 2014. Samples were collected within 100 ft. of 40 degrees, 12’, 37.8” N; 74 degrees, 31’, 37.2” W. Permission to sample at the site was first granted by Mr. Raymond Porutski and then by Mr. Peter Winkler, NJ Division of Fish & Wildlife, Bureau of Land Management, Assunpink Wildlife Management Area.

Soil samples, ~ 30 g, were mixed with 500 mL DI water to prepare a homogenized slurry. Aliquots (20 ml) of the slurry were then used to inoculate a basal medium (180 ml) in 250-ml glass serum vials. The basal medium was an inorganic Fe(III)-NH_4_^+^ enrichment medium (iFeN) described below. The composition of the iFeN medium (pH 4.5, which was adjusted by adding dropwise HCl) consisted of 177 mg/l NH_4_Cl, 77.9 mg/l (NH_4_)_2_SO_4_, 19.8 mg/l NaHCO_3_, 71.0 mg/l KHCO_3_, 9.00 mg/l KH_2_PO4, 100 mg/l MgSO_4_•7H_2_O, and 60.0 mg/l CaCl_2_•2H_2_O, in addition to 1 mg/l of a trace element solution [4] and 1 mg/l of a vitamin solution (ATCC^®^ MD-VS^™^). Other media that were tested but were less successful are listed in the Supporting Information (S1 File). The cultures were then supplemented with 6-line ferrihydrite (Fe_2_O_3_•0.5H_2_O; prepared according to Cornell and Schwertmann [11]), to obtain a final amount of 30 mmol/l of Fe(III). Because the ferrihydrite was prepared from ferric nitrite, it contained traces of nitrate (2 to 3 mg/l of ferrihydrite slurry). Twenty-five replicate inoculations were done under anaerobic conditions in an anaerobic chamber and the vials were sealed with butyl rubber septa, five autoclaved controls were prepared at the same time. To achieve strictly anoxic conditions, the headspace of each incubation vial was vacuumed and then flushed with a N_2_/CO_2_ (80:20) mixture, which was repeated three times. The enrichment cultures were then kept in dark at 25°C. After the culture’s color changed from reddish to dark gray, indicating that Fe(III) from the added ferrihydrite was being reduced, 10% (v/v) of a culture where a dark gray color had developed, was transferred to another 180 ml of fresh medium, containing a fresh supply of the ferrihydrite. During the full incubation experiment (~1 year), this transfer procedure was carried out three to four times, depending on the time of noted color change in the vials. Samples were collected about every two weeks for Fe(III) and NH_4_^+^ analysis and stored at -20 °C prior to conducting the molecular biology analysis described below. The enrichment cultures with the highest Fe(III) reduction and NH_4_^+^ oxidation were selected for the microbial community analysis via 16S rRNA gene sequencing on an Illumina MiSeq platform as described below.

### Isolation and subculture of iron reducers with simultaneous ammonium oxidation under anaerobic conditions

One milliliter of the final enrichment culture that exhibited repeatable and the highest simultaneous Fe(III) reduction and NH_4_^+^ oxidation was sampled and diluted by a factor ranging from 10 to 10^4^, after which the diluents were streaked on agar (18 g/l) iFeN medium plates with fresh ferrihydrite overlaid, and three control treatments, with no Fe(III), no NH_4_+, or with Fe(III) and tryptone soy but no NH_4_+ were prepared (see S1 File). These agar plate cultures were then incubated for 1 to 2 weeks under room temperature in an anaerobic glove bag filled with a N_2_/CO_2_ (80:20) gas mixture. Then selected colonies were applied again on fresh agar plates (same medium) to propagate the cultures, and the procedure was repeated three times. Single colonies with iron reducing capability were identified through the color change when Fe(III) was reduced to Fe(II), and were selected and isolated. All preparations for the inoculation and isolation steps were carried out under sterilized conditions. Bacteria isolates from these plates were then routinely sub-cultured in vials containing a liquid iFeN medium with about 10 mmol/l ferrihydrite and 2 mmol/l NH_4_Cl, at a pH of 4.5. Triple autoclaved controls, and controls with either no Fe(III) or no NH_4_^+^ were run in parallel. The cultures were then incubated for 20 days under strictly anoxic conditions at room temperature. Subsamples were collected about every two days from these cultures. The pH as well as Fe(III) and NH_4_^+^ were measured immediately, and samples were then stored at -20 °C until further molecular biology analysis were conducted.

In order to track the NH_4_^+^ transformation pathway of the selected strains, additional incubations using 100 μM, ~98% ^15^N NH_4_Cl (Cambridge Isotope Laboratories, Tewksbury, MA, USA) were conducted in parallel under the same conditions as described above (see also S1 File).

### Phylogenetic and physiological analyses of the isolated strains

The 16S rRNA gene of all the isolated strains with both Fe(III) reduction and NH_4_^+^ oxidation activity in the liquid media were amplified via PCR using multiple bacterial universal primers (Table A in S1 File), under the following conditions: 5 min at 94 °C, 30 cycles of 1 min at 94 °C, 1 min at specific annealing temp., 1.5 min at 72 °C and a step of 10 min at 72 °C. All the amplified PCR products were then run and visualized on a 1.2% agarose gel electrophoresis with syber safe stain in E-gel system. All the amplified products were purified and sequenced by Genewiz Inc. The sequences were analyzed using a BLAST search of the GenBank database. Phylogenetic trees of the partial 16S rRNA gene sequences and nearly full-length reference sequences were constructed using the MEGA software [12]. Selected strains were also settled on glass slides and viewed using a phase-contrast microscope (Leitz Labolux, ×400) and a Hitachi S-520 scanning electron microscope for morphological analysis [13].

### Investigation of the strain’s characteristics

All strains that could reduce Fe(III) while oxidizing NH_4_^+^ oxidation simultaneously during the incubations were used for further analyses of their growth characteristics.

Various oxyhydroxides and dissolved Fe(III) species were tested as possible Fe(III) sources for the cultured strains, in addition to ferrihydrite. The media for the cultures were processed as described before but supplemented with one of the alternative Fe(III) sources instead of ferrihydrite. Iron oxyhydroxides tested included goethite [α-FeOOH; particle size, ca. 10 μm; prepared according to Cornell and Schwertmann [11], lepidocrocite (γ-FeOOH; particle size, <250 μm; Alfa Aesar), hematite (α-Fe_2_O_3_; particle size, ca. 10 μm; Wako), or magnetite (Fe_3_O_4_; particle size, 20–30 nm; Alfa Aesar), to obtain a final amount of 30 mmol/l for ferrihydrite, goethite, and lepidocrocite, or 10 mmol/l for hematite and magnetite. The dissolved Fe(III) sources were ferric chloride, ferric citrate, and ferric EDTA, all with a final concentration of 10 mmol/l.

CO_2_ gas, sodium bicarbonate, sodium acetate, sodium succinate, and glucose were selected as potential carbon sources for the strains to conduct the Feammox reaction under otherwise identical growth conditions. Additional parallel incubations were also conducted using 20% ^13^C-CO_2_ in the headspace, 99 at. % ^13^C, < 3 atom % ^18^O (Cambridge Isotope Laboratories, Tewksbury, MA, USA) with 80% non-label CO_2_ gas.

It has been shown by many researchers that electron shuttles can in many instances increase the rate of iron reduction for iron reducing bacteria on a suite of iron oxides and oxyhydroxides [14–17]. Quinones are moieties that act as electron acceptors in the reduction of humic substances [18], and AQDS (9,10-anthraquinone-2,6-disulfonic acid) is quinone that has been widely used as a functional analogue to naturally-occurring humics to enhance iron reduction in laboratory incubations. Hence, we supplemented selected incubations with AQDS (25 μmol/l) to assess the impact of an electron shuttling compound on the iron-reducing and NH_4_^+^ removal capability of the selected strain.

### Hydrogen as possible electron donor for the isolated strain

Hydrogen (H_2_) was tested in two separate experiments, as an alternate electron donor for the isolated strain to grow on. In these experiments, the strain that was identified in the previous incubations as oxidizing NH_4_^+^ while reducing iron were grown in both the solid and liquid inorganic Fe(III) medium (iFe) without NH_4_^+^, but in the presence of H_2_ in the headspace. For this purpose, all cultures were incubated and sparged continuously with a N_2_/H_2_ (80%/20%) gas mixture in a bench top anaerobic glove bag. Changes in dissolved/extractable iron, and organic/inorganic carbon were monitored every two days during these incubations. All colonies and cultures were sampled every four days and stored at -20 °C for follow-up molecular biology analyses.

### Chemical analytical methods

An extraction of the slurries with 0.5N HCl was conducted for 24 hours at room temperature to determine their acid-extractable Fe(III), Fe(II), and NH_4_^+^ concentrations. Fe(II) concentration was measured spectrophotometrically with ferrizone at 560 nm [19]. For Fe(II) concentration measurements, the samples were immediately transferred to 25% (v/v) HCl solution to avoid Fe(II) oxidation. Extractable Fe(III) was evaluated as the difference between the extracted Fe(II) concentrations after and before reduction with excess hydroxylamine hydrochloride. Culture samples were centrifuged at 4000 rpm for 10 min to allow for chemical analysis of dissolved constituents. Dissolved inorganic nitrogen was analyzed using a Dionex^™^ Ion Chromatograph (LC3000) with an AS-22 and CS-16 column. Concentrations of organic and inorganic carbon were analyzed using a Shimadzu TOC-5000(A) analyzer. The N and C isotope measurements were done using a Finnigan MAT 253 mass spectrometer (Thermo Fisher Scientific, US) and the detailed methodology and calculations are described in the Supporting Information (S1 File).

### Molecular biology analytical methods

On day 0, 56, 172, and 300, of the incubations, nucleic acids were extracted from the subsamples of the enrichment batch cultures with the highest Fe(III) reduction and NH_4_^+^ removal. Nucleic acids were also extracted from all the strains that showed both Fe(III) reduction and NH_4_^+^ oxidation activity after being transferred to the liquid iFeN medium. DNA was extracted using a FastDNA^®^ spin kit for soil (MP Biomedicals, USA) as described by the manufacturer. RNA was removed from the total DNA extracts via RNase (Type II-A; Sigma-Aldrich) digestion.

16S rRNA gene sequencing was performed on the Illumina MiSeq platform for the enrichment samples. Domain-specific primers, targeting the V4 region of the 16S rRNA gene of bacteria, were amplified by primer-set 515F-806R following methods suggested by Caporaso *et al*. [20]. All PCR reactions were carried out with a Phusion^®^ High-Fidelity PCR Master Mix (New England Biolabs), and the PCR products were quantified and purified before sequencing. Sequencing libraries were generated using TruSeq^®^ DNA PCR-Free Sample Preparation Kit (Illumina, USA) following the manufacturer’s recommendations and index codes were added. The library quality was assessed on the Qubit@ 2.0 Fluorometer (Thermo Scientific) and Agilent Bioanalyzer 2100 system. At last, the library was sequenced on an Illumina MiSeq platform and about 250 bp paired-end reads were generated. All amplicon sequencing was conducted on an Illumina MiSeq platform at Novogene Co., Beijing, China.

A total of 17,750 sequences were obtained from the 16S rRNA gene each sample. Sequences analysis were performed using the Uparse software (Uparse v7.0.100, http://drive5.com/uparse/) [21]. Sequences with ≥97% similarity were assigned to the same OTUs, which resulted in 2025 Operational Taxonomic Units (OTUs). A representative sequence for each OTU was screened for further annotation.

## Results and discussions

### Identification of microbial enrichment cultures

During the full incubation experiment (~1 year) of the soil samples from the Assumpink site, which included three to four transfer procedures, 16 of a total of 25 enrichment cultures showed repeatable simultaneous Fe(III) reduction and NH_4_^+^ oxidation. The highest Fe(III) reduction and NH_4_^+^ oxidation was 81% and 65% respectively in the same culture during one incubation period. A fine-scale phylogenetic characterization of the enrichment cultures with the highest Fe(III) reduction and NH_4_^+^ oxidation, during the incubation was carried out via 16S rRNA gene sequencing on an Illumina MiSeq platform (Figure A in S1 File). A class of unidentified *Actinobacteria* as well as *Betaproteobacteria* were dominant during the incubations. The unidentified *Actinobacteria* increased from 6.0% to 59.2%, and *Betaproteobacteria* decreased from 43.2% to 32.6% of the total population after 300 days of incubation (Figure A in S1 File). The denitrifiers *Burkholderia* and *Ralstonia*[22], *which* belong to *Betaproteobacteria* accounted for 12.7% and 16.8% of the total bacterial number at the end of the one-year incubation period, indicating that sufficient NO_2_^-^/NO_3_^-^ was generated from the NH_4_^+^ oxidation for the growth of denitrifiers. Known iron reducers like *Geothrix fermentans* [23] and *Desulfovibrio* [24] were still found in the enrichment cultures at the end of the one-year incubation experiments, whereas *Geobacter* decreased from 9.2% to undetectable over this time and a group of unidentified *Acidimicrobiaceae* increased from 7.5% to 47.6% in these enrichment cultures during the sequential incubations.

### Isolation and characterization of the new strain, Acidimicrobiaceae sp. A6

As described above for the enrichment culture, a group of unidentified *Acidimicrobiaceae* increased substantially and became the dominant organisms after the one-year incubation. This final enrichment culture was then used to inoculate agar media plates in order to isolate the bacteria.

**Fig 1:**
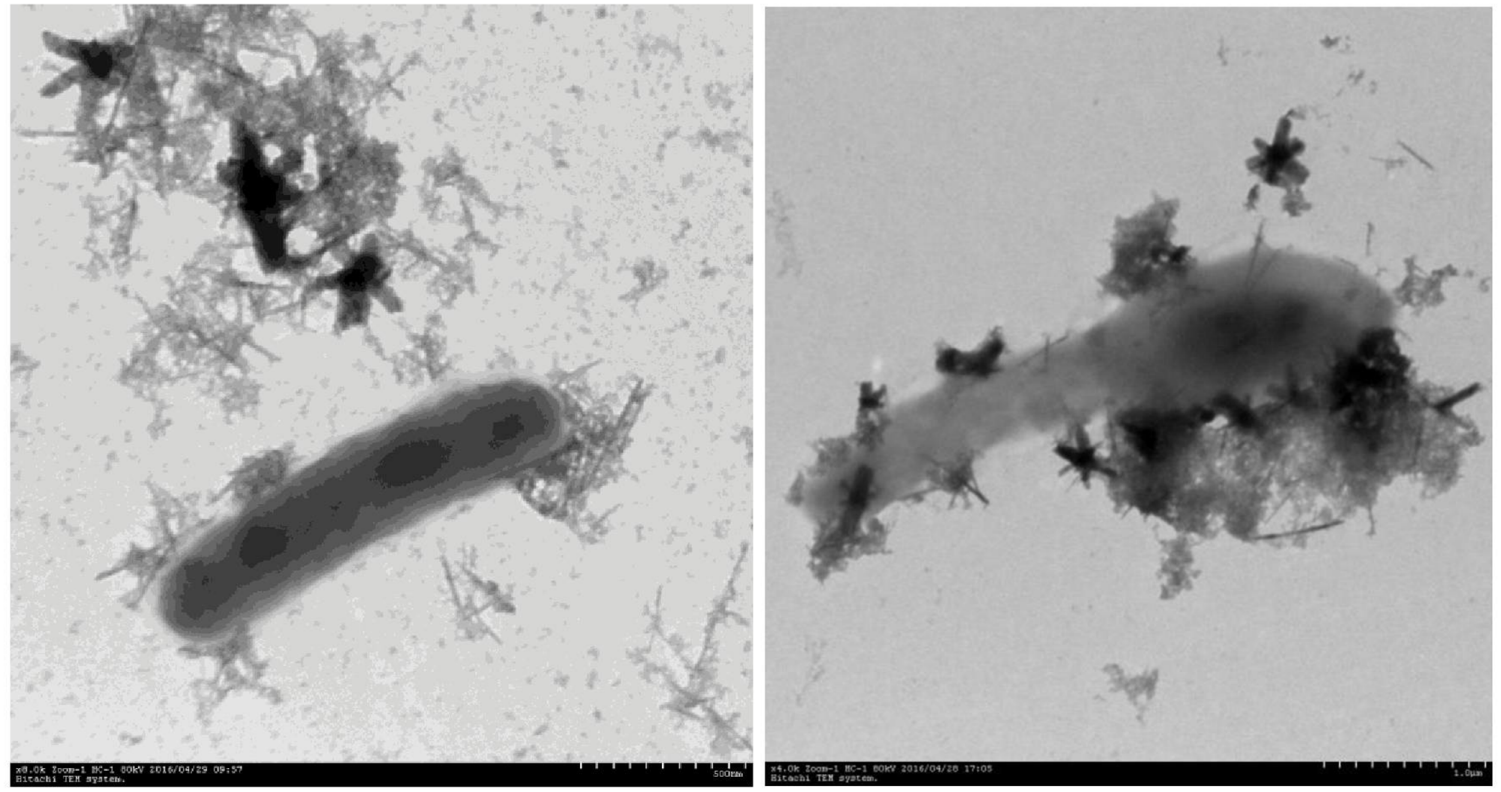
SEM image of strain A6 in an acidic, iron reducing, and ammonia oxidizing culture (iFeN).

After inoculating the agar medium plates and incubating them under anoxic conditions for 1 to 2 weeks, colonies with dark gray spots [representing the accumulation of ferrous iron, Fe(II)] were observed. No dark gray spots were found in the controls without Fe(III) or NH_4_^+^. A total of 113 colonies that could reduce Fe(III) to Fe(II) in the iFeN agar plates under anoxic conditions were picked from 4 replicate plates. Colonies were then transfer into liquid iFeN media, 22 cultures showed detectable and continuous NH_4_^+^ oxidation as well as iron reduction during 20 days of anaerobic incubation. Colonies grown on tryptone soy media were also transfer in the liquid iFeN media, however, no NH_4_^+^ oxidation was detected. After their 16S rRNA was amplified and sequenced with universal primers, these 22 strains were shown to be the same *Acidimicrobiaceae*-like strain. The *Acidimicrobiaceae* colonies on plates were small (diameter < 2mm) and visible only after 2 weeks of incubation. Microscopic examination of these colonies in the solid medium showed that the bacteria were gram-positive, and the cells were rod-shaped, 1.5–3 μm long by 0.5 μm wide (Fig 1).

**Fig 2:**
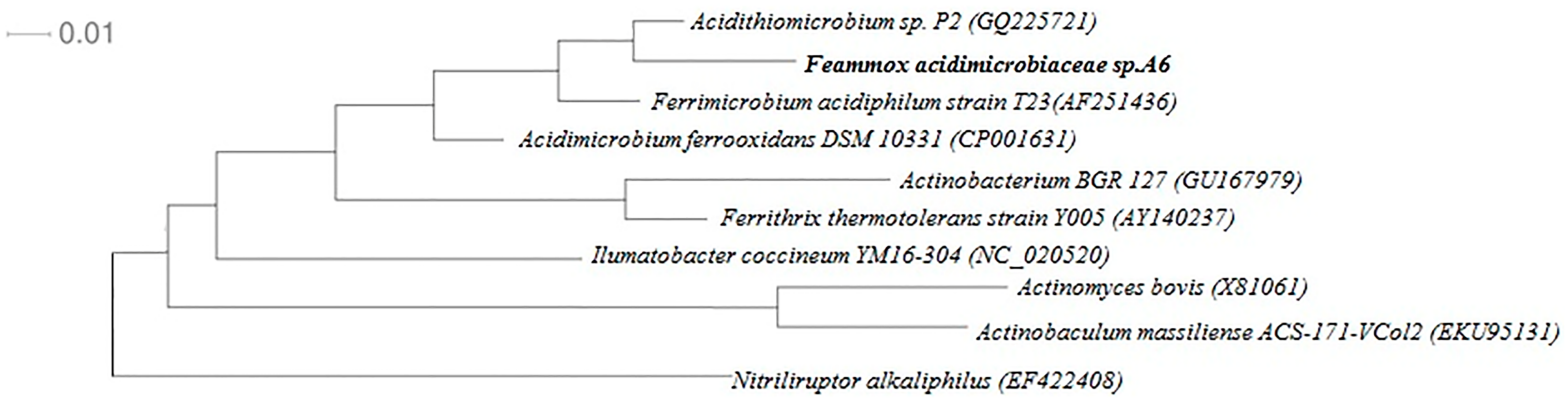
Phylogenetic tree derived from neighbour-joining analysis of partial 16S rRNA sequences. The tree was constructed with 16S rRNA gene sequences from strain A6 (MG589453) and from other bacteria of the *Acidimicrobiaceae* family. Sequences determined in this study are in bold. Bootstrap values were based on 1000 replicates each and are shown at the nodes with >50% bootstrap support. The scale bar represents 1% sequence divergence.

Approximately 1450 bp of 16S rRNA sequences were obtained via PCR and sequencing for this strain. A phylogenetic tree was reconstructed based on the 16S rRNA gene sequence of the isolated strain and other phylogenetically related strains (Fig 2). Per the phylogenetic analysis, this strain belongs to the *Acidimicrobiaceae* family and shows the highest similarity to *Ferrimicrobium acidiphilum* (with 92.3% identity), followed by *Acidimicrobium ferrooxidans* (with 90.2% identity) (Fig 2). These results reveal that the *Acidimicrobiaceae* strain isolated in this study represents a new strain, for which the name *Acidimicrobiaceae* sp. A6 is proposed, with the type strain A6 (ATCC, PTA-122488). The partial 16S rRNA gene of strain A6 was submitted to GenBank databases under the accession number MG589453.

### Electron donors and acceptors for the feammox reaction conducted by Acidimicrobiaceae sp. A6

The effects of different substrates on strain A6 were investigated. During incubation in the liquid iFeN medium, cultures of the *Acidimicrobiaceae* sp. A6 became increasingly turbid and dark gray-colored (due to the accumulation of ferrous iron). Their Feammox activity was verified by tracking NH_4_^+^ oxidation and iron reduction during the incubations. Bacterial numbers were quantified via qPCR, and the doubling time of the bacteria was determined from the time for the initial number of copies to double. Under the incubation conditions (pH = 4.5, temperature 25 °C, strictly anoxic, in an inorganic iFeN media with 25 μM of AQDS), after inoculating the liquid media with 1×10^3^−10^5^ copies of *Acidimicrobiaceae* sp. A6 per mL, the culture-doubling time of strain A6 was 8–10 days.

**Fig 3:**
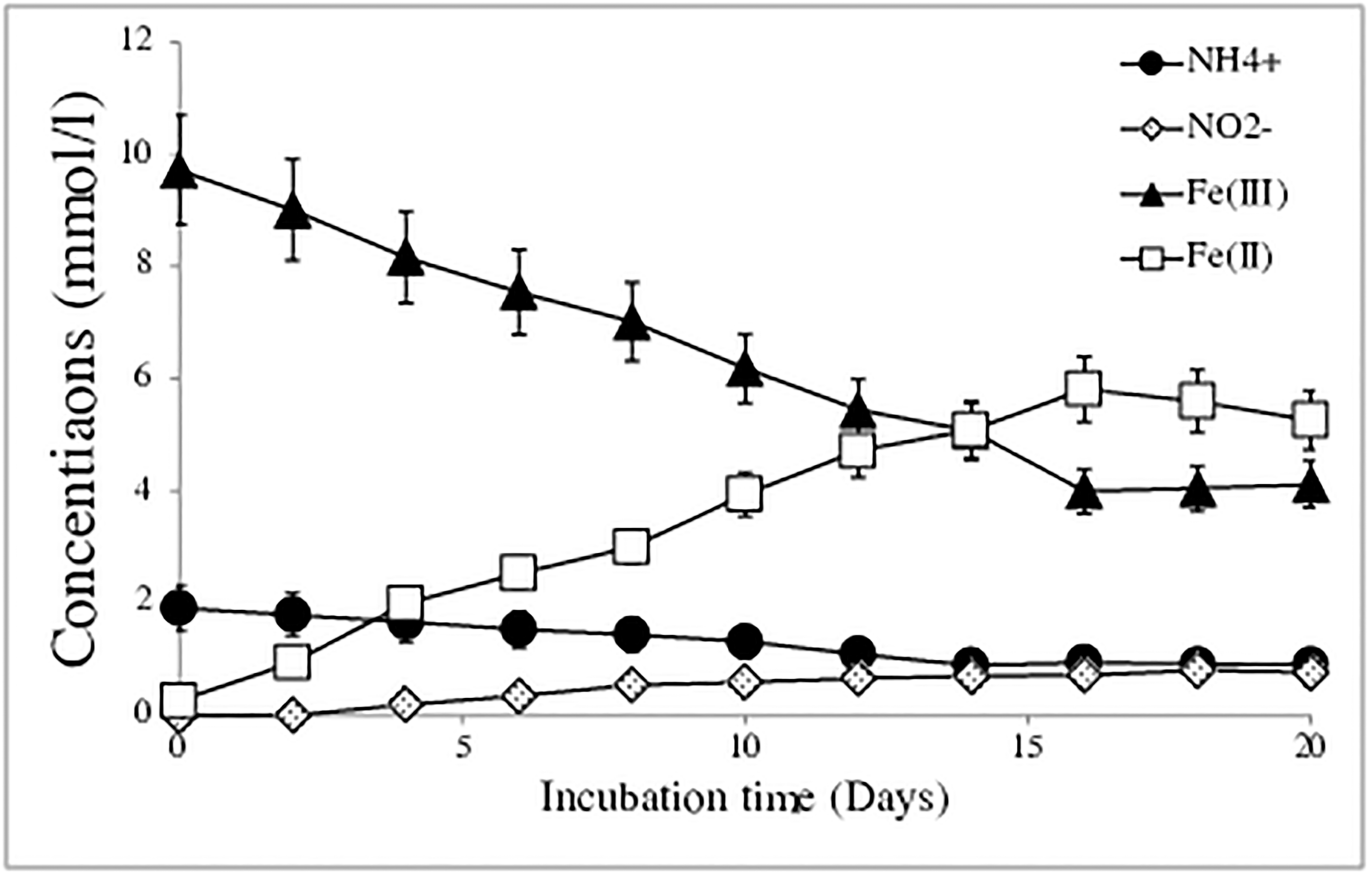
Concentration of NH_4_^+^, NO_2_^-^, Fe(II), and Fe(III) over 20 days of incubation in the liquid iFeN media with *Acidimicrobiaceae* sp. A6. The values represent the mean and standard error (n = 3).

With an initial copy number of 1×10^5^ per mL of *Acidimicrobiaceae* sp. A6 (diluted from a known concentration culture), 1.15 mmol/l of NH_4_^+^ were oxidized and 5.65 mmol/l of Fe(II) as well as 0.772 mmol/l of NO_2_^-^ were produced over 20 days of incubation (Fig 3). Controls without NH_4_^+^ resulted in no Fe(II) production, and controls without Fe(III) resulted in no NH_4_^+^ removal (Figure B in S1 File). Since no pH buffer was added to the system, the pH increased from 4.5 to 5 during these incubations. The observed ratio of NH_4_^+^-oxidized to Fe(II)-produced was 1:572 ± 0.05 (Figure C in S1 File), which is slightly lower than the 1:6 stoichiometry shown in Eq 1. When in addition to the above, 1g/l ^15^N-NH_4_Cl was added to parallel incubations as the only NH_4_^+^ source, an increase of 5.08% δ^15^N- NO_2_^-^ was measured after a 48-hour incubation, while no ^15^N-N_2_ was detected, showing that NO_2_^-^ is the main product of the NH_4_^+^ oxidation in this Feammox reaction with strain A6 (Table B in S1 File).

Members of family *Acidimicrobiaceae* are known to participate in the iron cycle by either reducing or oxidizing iron. At present, this family is subdivided into the following separate genera: *Acidimicrobium ferrooxidans* [25], *Aciditerrimonas ferrireducens* [26], *Ferrimicrobium acidiphilum* [27], and *Ferrithrix thermotolerans* [27]. *Acidimicrobium ferrooxidans* is an iron oxidizer in acidophilic soil environments and capable to fix CO_2_ for autotrophic growth [28]. Two acidophilic iron-reducers, *Ferrimicrobium acidiphilum* and *Ferrithrix thermotolerans* have been isolated, one from a mine site in North Wales, UK (isolate T23), and the other one from a geothermal site in Yellowstone National Park, Wyoming, USA (Y005) [27]. Both isolates can catalyze the dissimilatory reduction of ferric iron, using glycerol as electron donor in an oxygen-free medium, and can also oxidize iron under certain conditions [27]. *Aciditerrimonas ferrireducens* has been grown anaerobically conducting dissimilatory reduction of ferric iron at an optimum temperature of 50 °C, while no oxidation of ferrous iron was observed when this bacterium was exposed to oxygen [26, 29]. However, none of these *Acidimicrobiaceae* has been reported to use NH_4_^+^ as electron donor for Fe(III) reduction until the isolation of this *Acidimicrobiaceae* sp. A6 strain.

### Iron-reduction and ammonium oxidation of strain A6 under different environmental conditions

#### Effect of pH

**Fig 4:**
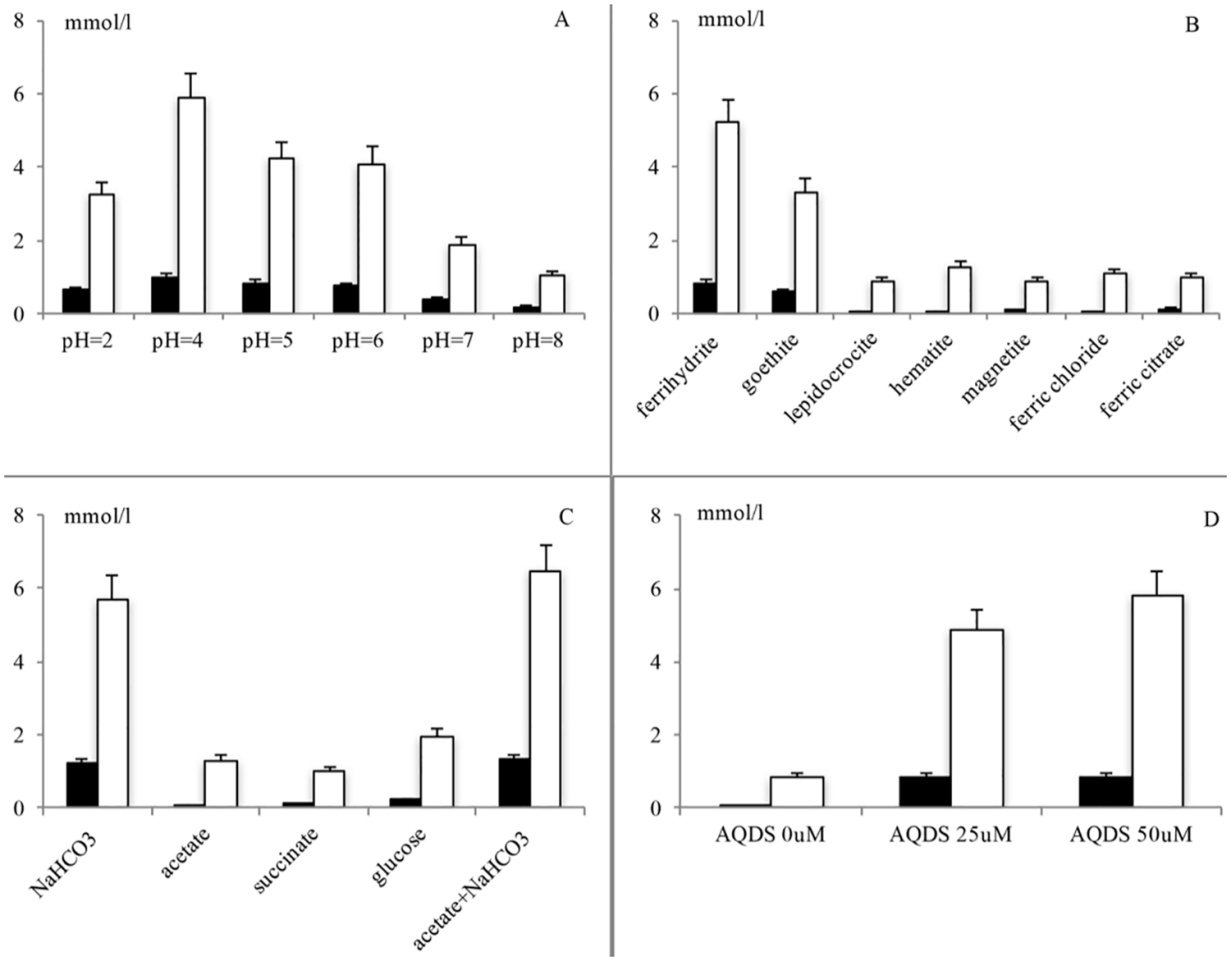
Amount of NH_4_^+^ removal (black) and Fe(III) reduction (white) over 14 days of incubation with *Acidimicrobiaceae* sp. A6. Under different (a) pH values, (b) iron source, (c) carbon source, and (d) AQDS concentions. The values represent the mean and standard error (n = 3).

The growth of various iron-reducing bacteria can occur over a wide range of pH values[30–32]. As shown by Eq 1, one would expect that the Feammox process requires acidic conditions, and ΔG becomes positive for alkaline conditions. To further investigate the effect of pH on the strain A6, its Feammox activity in terms of the iron reduction and NH_4_^+^ oxidation capabilities was studied at a pH of 2.0, 4.0, 5.0, 6.0, 7.0 and 8.0 (Fig 4A). In these incubations, the initial total Fe(III) measured was ranged from 9.12 to 11.6 mmol/l and the initial NH_4_^+^ concentration from 2.03 to 2.17 mmol/l. After 14 days of incubation, the maximum amount of iron reduced over the pH range studied occurred at a pH = 4.0, which is shown in Fig 4A. These results are consistent with our previous study showing that ammonium oxidation under iron-reducing conditions is favored under weakly acidic conditions [31].

The removal of NH_4_^+^ followed similar patterns as the Fe(III) reduction. As shown in Fig 4A, during 14 days of incubation, the amount of NH_4_^+^ oxidized was highest in the incubations with a pH = 4 (1.13 mmol/l). The amounts of NH_4_^+^ oxidized are impressive considering there was no measurable oxygen or any initial NO_3_^-^/NO_2_^-^ in the cultures. Based on the Fe(III) reduction and NH_4_^+^ removal, the optimal pH in this study for strain A6 was 4.0. This pH is close to that of the New Jersey wetland soil (pH 4~4.5) where the Feammox reaction was first described and soil samples for this study were collected.

#### Effect of iron source

Anaerobic NH_4_^+^ oxidation reaction and the growth of strain A6 was only observed in cultures to which either 6-line ferrihydrite or goethite was added. No detectable NH_4_^+^ oxidation was observed in samples with lepidocrocite, hematite, magnetite, ferric chloride, or ferric citrate as the Fe(III) source (Fig 4B).

#### Effect of carbon source

Strain A6 could grow in both organic carbon-free solid and liquid media. After 14 days of incubation amended with ^13^C labeled CO_2_, the ^13^C measured in cells of Strain A6 increased to 10.3%, while no ^13^C was detectable in the autoclaved control (Table C in S1 File). Growth of isolate A6 showed no difference in media containing NaHCO_3_ and augmented with acetate, sodium succinate, or glucose, as compared to control cultures to which only NaHCO_3_ was added. Attempts to grow strain A6 in the liquid medium with only sodium acetate, sodium succinate, glucose (without inorganic carbon) as the carbon source were unsuccessful, and the NH_4_^+^ oxidation was negligible without the addition of inorganic carbon (Fig 4C), all of which indicates that A6 requires inorganic carbon to grow.

#### Effect of electron shuttling compounds

When colonies were transferred to the liquid inorganic NH_4_^+^-Ferric iron medium without AQDS, no growth was observed in the liquid medium, while strain A6 could grow, oxidize NH_4_^+^, and reduce Fe(III) in the presence of 25 μM and 50 μM of AQDS (Fig 4D).

#### Effect of H_2_ on growth of A6 and its oxidation of ammonium

Incubations of strain A6 were conducted for 14 days on plates that were continuously sparged with a N_2_/H_2_ gas mixture (80%/20%) in an anaerobic glove bag, resulting in ~ 1.13 mmol/l of H_2_ in the headspace. *Acidimicrobiaceae* sp. A6 colonies were found to be growing without NH_4_^+^ in the agar (18g/l) ferric medium plates (iFeo) after 14 days of incubation in the presence of H_2_. The control samples in the same agar iFeo medium plate but under H_2_-free conditions did not exhibit any growth of strain A6, nor any production of Fe(II). A6 cultures were also transferred from these agar iFeo medium plates to a liquid ferric medium without NH_4_^+^ (iFeo) in a 200-mL glass bottle with a N_2_/H_2_ gas mixture (2–5% H_2_) in the headspace and absence of NH_4_^+^. After 4 weeks of incubation, 4.27 mmol/l Fe(II) was produced, while no Fe(III) reduction was detected in the sterile controls. These results indicate A6 can grow on H_2_ as the electron donor instead of NH_4_^+^

In the second H_2_ incubation experiment, A6 cultures were transferred first to the same iFeo medium in a 200-mL glass bottle with a N_2_/H_2_ gas mixture (2–5% H_2_) in the headspace. A total of 3.58 mmol/l Fe(II) was produced after 21 days in these incubations. These A6 cultures were then transferred back to the inorganic ferric iron, NH_4_Cl (‘iFeN’) media and incubated as described under section 2.3 with or without H_2_ for 21 days. However, no NH_4_^+^ oxidation was observed in any of these cultures, and Fe(III) reduction was detected only in the cultures incubated with H_2_. This indicates that the strain may have a complex regulatory system that may not be able to seamlessly switch modes of using NH_4_^+^ and H_2_ as electron acceptor.

## Conclusions

The new strain *Acidimicrobiaceae* sp. A6 was isolated from riparian wetland soils in New Jersey, and it was shown that this strain can oxidize NH_4_^+^ while reducing Fe(III) under anaerobic conditions, utilizing CO_2_ as its carbon source. Of the Fe(III) sources examined, ferrihydrite resulted in the highest NH_4_^+^ oxidation during the incubations. More as well as faster NH_4_^+^ oxidation was observed at a pH of 4 than at the other pH values examined. The presence of an electron shuttling compound (AQDS in this case) was required to grow the pure A6 strain in a liquid medium with ferrihydrite as the terminal electron acceptor.

Whereas A6 remained dominant in a Feammox enrichment culture and was stable for over a year, the pure A6 strain remained active for only one to two months, indicating that important co-factor/s were missing in our incubations that are required to maintain strain A6 active over longer time periods.

## Supporting information

S1 File Supporting information: Supplemental methods, materials, and results. (DOCX)

S2 File Chemical analyses data. (XLSX)
