# Supplementary material for: Isolation and characterization of an ammonium-oxidizing iron reducer: Acidimicrobiaceae sp. A6
Source: PLoS One. 2018 Apr 11;13(4):e0194007. doi: 10.1371/journal.pone.0194007 (PMC5894979; doi:10.1371/journal.pone.0194007)
Supplement: S1 File — (DOCX) [file pone.0194007.s001.docx]

**Isolation and Characterization of the autotrophic, ammonium-oxidizing, iron-reducing, acidophilic: Feammox *Acidimicrobiaceae sp. A6***

Shan Huang and Peter R. Jaffé*

**Supporting Information.**

**1. Supplemental Methods**

*1.1 Agar iFeN medium for strain isolation*

The composition of a basal medium consisted of 107 mg/l NH_4_Cl, 71.0 mg/l, KHCO_3_, 7.2 mg/l NaCl, 9.6 mg/l Na_2_SO_4_, 19.8 mg/l NaHCO_3_, 9.00 mg/l KH_2_PO4, 100 mg/l MgSO_4_•7H_2_O, and 60.0 mg/l CaCl_2_•2H_2_O, in addition to 1 mg/l of a trace element solution [1], and 1 mg/l of a vitamin solution (ATCC® MD-VS™). Rare earth metals mines were also added to a subset of the *iFeN* medium, but without detectable effect on the strain’s growth. Agar was added in these media with a final concentration 18g/l. To avoid that the iron supplement settled on the bottom of the plates, the agar media was first allowed to solidify, and then 0.5 ml of 5 mg/l 6-line ferrihydrite was gently spread on the top of the agar plates. The agar ferric iron, NH_4_Cl media (‘iFeN’) contained about 3 mmol/l ferrihydrite and 2 mmol/l NH_4_Cl. Three control agar plates were prepared, media with 2 mmol/l NH_4_Cl but no Fe(III), media that contained 3 mmol/l ferrihydrite but no NH_4_^+^, and only Fe(III) media added with 2 mmol/l tryptone soy (TSA) as a possible carbon source, were conducted under the same incubation conditions. All the agar plate cultures were then incubated for 1 to 2 weeks under room temperature in an anaerobic glove bag filled with a N_2_/CO_2_ (80:20) gas mixture. Random selected colonies were applied on the same fresh agar medium plates again to propagate the cultures, and the procedure was repeated three times. Single colonies with iron reducing capability were identified through the color change when Fe(III) was reduced to Fe(II), and were selected and isolated.

*1.2 Isotopic Tracer Incubations with ^15^NH_4_Cl addition*

Incubations of *Acidimicrobiaceae* sp. A6 combined with ^15^NH_4_^+^ were conducted in an anaerobic glovebox to track the nitrogen species during the Feammox process, following a slightly modified procedure discussed elsewhere [2, 3]. Briefly, as a parallel incubation to that described in section 2.2, 20 mL vials were divided into the two following additional treatments: (1) sterile anoxic DI water instead of ^15^NH_4_Cl (control), and (2) ^15^NH_4_Cl addition (^15^N at 99.0%, Cambridge Isotope Laboratories, Inc., Tewksbury, MA, ^15^NH_4_^+^).

The vials with the ^15^NH_4_^+^ treatment were spiked with 1.0 mL of stock solution of ^15^NH_4_Cl, purged with high purity Helium, through the septa to achieve a dry weight of 20 mg ^15^N [2, 4]. All vials were vigorously shaken to homogenize the solutions before incubation. Since no ^15^NO_3_^-^ was detected during the incubation, only the ^15^N- isotope value (δ^15^N) of NO_2_^-^ was analysis in this isotopic tracer experiment. δ^15^N of NO_2_^-^ was determined according to Böhlke et al. (2007)[5]. Briefly, after 48 h of incubation, NO_2_^-^ was first reduced to N_2_O by selected denitrifiers. 5 mL of gas sample were then collected using gastight syringes and then injected into 12 mL pre-evacuated serum vials sealed with butyl-rubber septa. N_2_O was concentrated and purified on a GasBench II and δ^15^N was analyzed on a Finnigan mat 253 mass spectrometer (Thermo Fisher Scientific, US)[6]. The headspace N_2_O concentrations were analyzed on a Shimadzu GC-2014 equipped with an ECD. Five replicate samples were conducted.

*1.3 Isotopic Tracer Incubations with ^13^CO_2_ addition*

To determine if the culture is capable of assimilating inorganic carbon, ^13^C isotopic incubations were conducted to track the flow of CO_2_ fixed into biomass and DNA, and to evaluate the autotrophic ability of strain A6. For this purpose, 20 mL of A6 culture (about 10^5^ cells/l) were put into sterilized 200 mL serum bottles with butyl rubber stoppers inside a glove box containing a N_2_ atmosphere. These cultures were incubated under anoxic conditions with 99% ^13^CO_2_ (Cambridge Isotope Laboratories, Tewksbury, MA, USA) in the 50-ml headspace. In order to simulate the biological activity of strain A6 and to maintain a constant label, the headspace of every sample was removed and renewed every 3 days. The samples were incubated for 14 days at room temperature.

After 14 days of incubation, the vials were opened in a glove box under N_2_, and microbial biomass was extracted using a chloroform fumigation extraction (CFE) [7]. CFE extracts microbial biomass C by lysing the cells with chloroform and releasing the products of cell lysis into a salt solution as dissolved organic carbon (DOC). The concentration of ^13^C in the dissolved microbial biomass and their stable carbon isotope ratio were determined using a Finnigan mat 253 mass spectrometer (Thermo Fisher Scientific, US).

**2.** **Supplemental Results** Table S1. PCR primers used for the amplification in this study

| Primer | Sequence (5’-3’) | Annealing temp. |
| --- | --- | --- |
| 27f | GACAAACTTCGCAGCGG | 57 |
| 341f | CCTAYGGGRBGCASCAG | 57 |
| 519r | GWATTACCGCGGCKGCTG | 57 |
| 806r | GGACTACNNGGGTATCTAAT | 57 |
| 1392r | ACGGGGCGGTGTGTAC | 59 |
| 1492r | TCACCCAGGACGCTGTTC | 59 |

Table S2. Changes in ^15^N- isotope value of NO_2_^-^ in the Feammox *Acidimicrobiaceae* sp. A6 cultures after 48 hours of incubation with ^15^NH_4_^+^ treatments.

|  | Control | ^15^NH_4_Cl addition |
| --- | --- | --- |
|  | δ^15^N- NO_2_^-^ | δ^15^N- NO_2_^-^ |
| 0 hour | -0.94% | -1.51% |
| 48 hours | -1.03% | 3.57% |

Table S3. Ratio of ^12^C and ^13^C in cells of Feammox *Acidimicrobiaceae* sp. A6 during 14 days of incubation after adding ^13^C labeled CO_2_ (50 of headspace in 120 ml vials)

|  | Cell (with methanol wash) | |
| --- | --- | --- |
|  | ^12^C | ^13^C |
| Day 0 | 98.2% | 1.80% |
| Day 14 | 89.7% | 10.3% |

Figure S1. Microbial community heat map analysis of the enrichment cultures with the highest Fe(III) reduction and NH_4_^+^ oxidation on day 0, 56, 172, and 300 of the incubation

**Day 0**

**Day 57**

**Day172**

**Day 300**

Figure S2. Amount of NH_4_^+^ removal (black) and Fe(III) reduction (white) over 14 days of incubation with *Acidimicrobiaceae* sp. A6. under different control conditons. The values represent the mean and standard error (n=3).

**
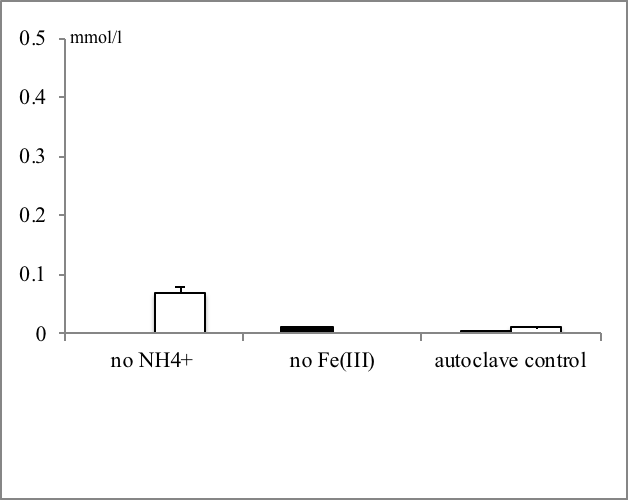
**

Figure S3. Ratio of Fe(II) produced to NH_4_^+^ removed over 20 days of incubation in the liquid iFeN media with *Acidimicrobiaceae sp*. A6. The values represent the mean and standard error (n=3).

**References**

1. Huang S, Jaffe PR (2015) Characterization of incubation experiments and development of an enrichment culture capable of ammonium oxidation under iron-reducing conditions. Biogeosciences 12: 769-779. doi: 10.5194/bg-12-769-2015

2. Yang WH, Weber KA, Silver WL (2012) Nitrogen loss from soil through anaerobic ammonium oxidation coupled to iron reduction. Nat Geosci 5: 538-541. doi: 10.1038/Ngeo1530

3. Shrestha J, Rich JJ, Ehrenfeld JG, Jaffe PR (2009) Oxidation of Ammonium to Nitrite Under Iron-Reducing Conditions in Wetland Soils Laboratory, Field Demonstrations, and Push-Pull Rate Determination. Soil Sci 174: 156-164. doi: 10.1097/SS.0b013e3181988fbf

4. Ding LJ, An XL, Li S, Zhang GL, Zhu YG (2014) Nitrogen Loss through Anaerobic Ammonium Oxidation Coupled to Iron Reduction from Paddy Soils in a Chronosequence. Environmental science & technology 48: 10641-10647. doi: 10.1021/es503113s

5. Bohlke JK, Smith RL, Hannon JE (2007) Isotopic analysis of N and O in nitrite and nitrate by sequential selective bacterial reduction to N2O. Anal Chem 79: 5888-5895. doi: 10.1021/ac070176k

6. Dahnke K, Thamdrup B (2013) Nitrogen isotope dynamics and fractionation during sedimentary denitrification in Boknis Eck, Baltic Sea. Biogeosciences 10: 3079-3088. doi: 10.5194/bg-10-3079-2013
